# Pipeline to Design Inbred Lines and F1 Hybrids of Leaf Chicory (Radicchio) Using Male Sterility and Genotyping-by-Sequencing

**DOI:** 10.3390/plants12061242

**Published:** 2023-03-09

**Authors:** Francesco Scariolo, Fabio Palumbo, Silvia Farinati, Gianni Barcaccia

**Affiliations:** Department of Agronomy Food Natural resources Animals Environment, Campus of Agripolis, University of Padova, 35020 Legnaro, PD, Italy

**Keywords:** chicory, endive, RADseq, male sterility, molecular breeding, SNPs, inbred lines, F1 hybrids, predicted breeding value

## Abstract

Chicory, a horticultural crop cultivated worldwide, presents many botanical varieties and local biotypes. Among these, cultivars of the Italian radicchio group of the pure species *Cichorium intybus* L. and its interspecific hybrids with *Cichorium endivia* L.—as the “Red of Chioggia” biotype—includes several phenotypes. This study uses a pipeline to address the marker-assisted breeding of F1 hybrids: it presents the genotyping-by-sequencing results of four elite inbred lines using a RADseq approach and an original molecular assay based on CAPS markers for screening mutants with nuclear male sterility in the radicchio of Chioggia. A total of 2953 SNP-carrying RADtags were identified and used to compute the actual estimates of homozygosity and overall genetic similarity and uniformity of the populations, as well as to determine their genetic distinctiveness and differentiation. Molecular data were further used to investigate the genomic distribution of the RADtags among the two *Cichorium* species, allowing their mapping in 1131 and 1071 coding sequences in chicory and endive, respectively. Paralleling this, an assay to screen the genotype at the male sterility locus *Cims-1* was developed to discriminate wild-type and mutant alleles of the causative gene *myb80-like*. Moreover, a RADtag mapped close to this genomic region proved the potential application of this method for future marker-assisted selection tools. Finally, after combining the genotype information of the core collection, the best 10 individuals from each inbred line were selected to compute the observed genetic similarity as a measure of uniformity as well as the expected homozygosity and heterozygosity estimates scorable by the putative progenies derived from selfing (pollen parent) and full-sibling (seed parent) or pair-wise crossing (F1 hybrids). This predictive approach was conducted as a pilot study to understand the potential application of RADseq in the fine tuning of molecular marker-assisted breeding strategies aimed at the development of inbred lines and F1 hybrids in leaf chicory.

## 1. Introduction

**Figure 1:**
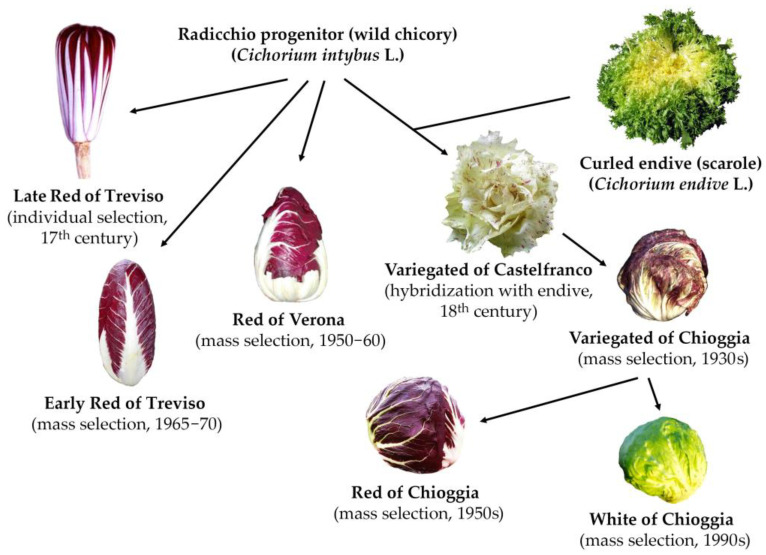
An overview on the main cultivated biotypes of Italian Radicchio, including the pure *C. intybus* forms Late Red of Treviso, Early Red of Treviso and Red of Verona, and the interspecific *C. intybus* × *C. endivia* hybrids Variegated of Castelfranco, Red of Chioggia and White of Chioggia, along with their phylogenetic relationships as reconstructed on the basis of historical and molecular data.

*Cichorium intybus* L., or chicory (2*x* = 2*n* = 18), is a perennial leafy vegetable belonging to the Asteraceae family. This species, cultivated worldwide for its adaptability to various environmental conditions, has been domesticated into current vegetable products. It is an economically important European horticultural crop; additionally, it is one of the most important among those cultivated in the Veneto region of Northeast Italy, where it is differentiated into multiple different biotypes. The phenotypic variability observable in this species is well-represented by the local varieties of *C. intybus* originally from the Veneto region, where this crop took the traditional name “radicchio” [1,2,3,4,5]. Multiple chicory varieties are cultivated in this area, where its common ancestral biotype was first introduced during the 17th century—the “Late Red of Treviso”. During the following centuries, other biotypes differentiated by morphological selection or due to interspecific hybridisation with its related interfertile species, namely, *Cichorium endivia* L., or endive (2*x* = 2*n* = 18) [3,5]. Within the *Cichorium* genus, the compatible and crossable species *C. endivia* is present, which is characterised by having a completely different mating habit from *C. intybus*. In the case of chicory, an efficient sporophytic self-incompatibility (SSI) system causes an almost obligate allogamous mating system that makes the development of highly homozygous inbred lines very difficult to achieve by selfing; however, it is possible to obtain by full-sibling strategies [4,6], whereas in the case of endive, a prevalently autogamous species, interplant pollination is more difficult to realize, thus inhibiting the easy obtainment of F1 hybrids by pair-wise crossing. Despite this, and as previously mentioned, it is possible to successfully cross these two *Cichorium* species for the development of interspecific hybrids and introgressant genotypes. An overview of the main cultivated biotypes of Italian radicchio—including pure *C. intybus* forms such as “Rosso di Treviso Tardivo” (Late Red of Treviso), “Rosso di Treviso Precoce” (Early Red of Treviso) and “Rosso di Verona” (Red of Verona), and interspecific *C. intybus* × *C. endivia* hybrids such as “Variegato di Castelfranco” (Variegated of Castelfranco), “Rosso di Chioggia” (Red of Chioggia) and “Bianco di Chioggia” (White of Chioggia), along with their phylogenetic relationships as reconstructed on the basis of historical and molecular data—are reported in Figure 1 (for details, see Basso et al. [5] and references therein).

In the past, by crossing these two species, biotypes of radicchio appeared, such as the “Red of Chioggia”, which now have high economic importance and traditional value and have become an irreplaceable genetic resource of phenotypic variability exploitable for future breeding improvement plans in this crop [3,5,7,8]. Moreover, nuclear male sterility (NMS) was found to be another efficient reproductive barrier observed in leaf chicory, particularly in the cultivated varieties of the “Red of Chioggia” biotype [9,10], capable of enhancing cross-pollination for the development of F1 hybrid seed stocks at a large scale. In particular, NMS in Red of Chioggia was found to be the result of a four nucleotide insertion (5′-AATT-3′) within the second exon of the *myb80-like* gene that, in the recessive homozygous state (*msms*), is responsible for the lack of pollen maturation within anthers [11]. The insertion causes a frameshift of the coding sequences, which is responsible for a shorter and nonfunctional MYB80-like protein due to the insurgence of a “stop” codon in between the second exon of the gene. Consequently, when in the recessive homozygous state, the male-sterile phenotype appears; its causative gene was shown to be characterised by full penetrance and expressivity [9,10].

These two reproductive barriers (SSI and NMS)—known for preventing or minimizing self-pollination, and hence, enhancing heterozygosity and, more generally, genetic diversity in natural populations—can be very useful or inhibitory in crop breeding depending on the aim. In fact, as they force out-breeding, thus reducing the frequency of homozygous progenies, these two reproductive barriers negatively influence the development of inbred parental lines; however, they can be positively adopted to direct pair-wise crossing for the constitution of F1 hybrids. In particular, SSI in chicory, which is reported to be incompletely functional allowing low ratios of inbreeding [12,13,14,15], normally inhibits self-pollination and the constitution of inbred lines; MS, while also preventing self-pollination, can be exploited to direct cross-pollination between two genetically differentiated parental lines using one as the pollen parent (male-fertile; *Ms/*–) and the other as the seed parent (male-sterile; *msms*), paternal and maternal genotypes, respectively. Adopting this breeding strategy, highly heterozygous F1 hybrid progenies can be obtained by avoiding the harvesting of seeds from selfing from the male-sterile maternal line. Given the characteristics and possible implications of these two reproductive barriers, the adoption of molecular tools for screening breeding populations to select the desired genotypes would improve the breeding strategies of *Cichorium* crops by reducing the time and costs needed to obtain the target plant phenotype.

While the first biotypes of radicchio were selected by phenotypic mass selection based on morphological traits and descriptors, the current breeding strategies are supported by genotypic selection assisted by molecular markers and genomics as the main analytical tools. Currently, the newly released varieties of pure *C. intybus,* as well as its interspecific biotypes with *C. endivia*, are mainly F1 hybrids developed by Italian or European seed firms through large-scale single crosses between inbred lines selected according to their specific combining ability [5,16]. Thus, to maximise the heterosis phenomenon [17]), breeding programs of radicchio have significantly improved in recent years thanks to the use of more efficient molecular tools and analytical platforms [16,18,19,20,21]. To date in chicory, several linkage maps saturated with DNA markers and spanning the entire genome size have been produced and are available [18,19,21,22]. In particular, 29 selected simple sequence repeat (SSR) markers have demonstrated their potential in determining genetic similarities and differences in chicory populations for distinctiveness, uniformity and stability (DUS) testing and plant variety protection (PVP) [16]. In addition to SSR markers, one phenotype-related molecular marker associated with NMS in chicory has been recently developed that is based on allele-specific PCR (AS-PCR) for *Myb80-like* gene genotyping [11].

Breeding projects assisted by molecular markers require informative, reliable and reproducible genotyping approaches (i.e., protocols that are technically transferable among laboratories). Additionally, the possibility of predicting a specific phenotype by characterising the genotype of one specific locus can greatly help and speed the development of new varieties or the preservation or implementation of specific phenotypes. Currently, multiple genomic tools can be used [23], and the cost-effective aspect has become fundamental in the choice of which method to adopt for genotyping analyses. Among the various approaches that can be chosen, strategies based on genotyping-by-sequencing (GBS) have demonstrated their suitability for the practical applications previously mentioned. There are many GBS-related techniques that differ in the sequencing platform, the chemistry they are based on, the type of output, data throughput and other aspects [24,25,26,27,28,29]. Among the available GBS approaches, restriction-site associated DNA sequencing (RADseq) [30] has demonstrated its potential use in different crop species for breeding, PVP and traceability purposes [16,31,32]. Among the several advantages provided by the adoption of this approach is the unnecessity of an already sequenced genome because a comparative analysis between the sequenced reads can be made even without mapping them onto chromosomes. This certainly makes RADseq a suitable method for unstudied species and partially or poorly studied species, even if the availability of a representative genome is key helpful information for sequencing-based approaches.

**Figure 2:**
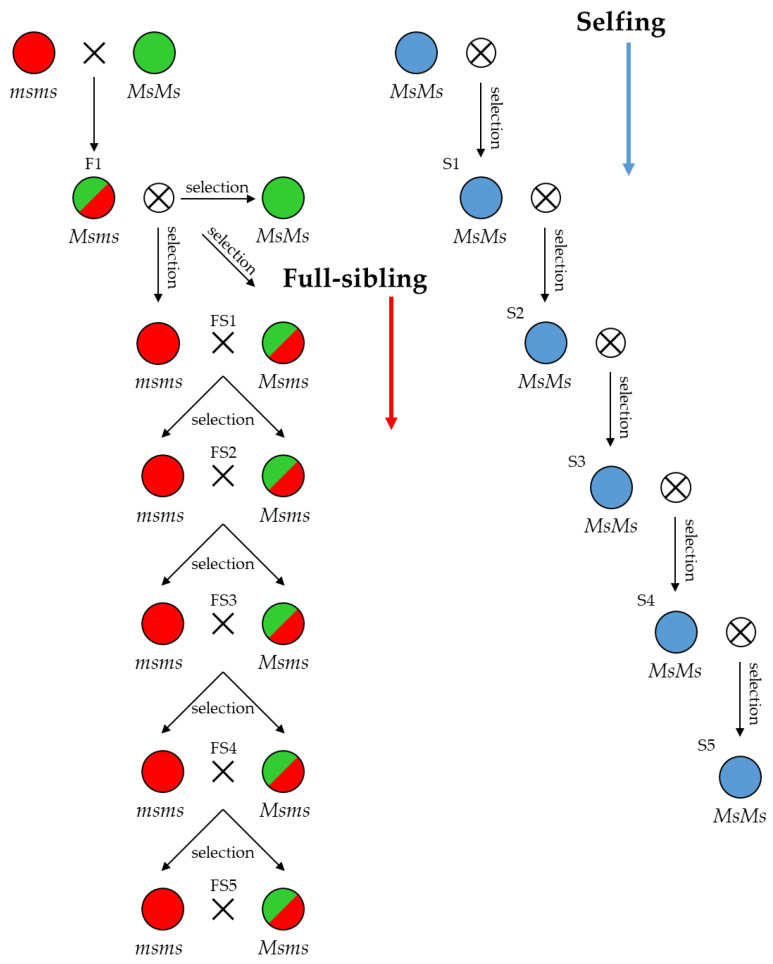
Breeding schemes exploiting male-sterile (*msms*) and male-fertile (*Ms/–*) genotypes in full-sibling and selfing strategies for inbred line development. FS: full sibling; S: selfing.

Therefore, the final goal of this study is to provide a screening protocol for genotyping these crops and their interspecific hybrids for breeding purposes by providing NGS-derived platforms, whose applications are faster and cheaper, and outputs which are more informative than the already available PCR-based markers for genotyping. In this study, taking advantage of the recently published genomes of both chicory and endive [33], we evaluated the technical potential and robustness of a RADseq approach as a strategy for GBS-related analysis. In this framework, RAD sequencing has been adopted for the molecular characterisation of four populations of the interspecific biotype “Red of Chioggia” of radicchio, which derives from an ancestral crossing of *C. intybus* and *C. endivia*. In detail, the aims were to verify the suitability of the RADseq method for genotyping this crop, to establish its ability to determine the genetic distinctiveness and uniformity of four full-sibling (FS) lines and to hypothesise the average heterozygosity and genetic similarity obtainable in the progenies derived from specific crosses or self-pollination in future breeding plans. A representation of the canonical breeding schemes exploiting the NMS locus for breeding full-sibling and selfing-derived inbred lines is available in Figure 2. Highly homozygous lines can be subsequently used in pair-wise crossing systems between highly dissimilar inbred lines to produce F1 hybrid seeds.

In addition, parallel to the GBS approach and given the nucleotide sequence of the 4 nt insertion (AATT) within the previously mentioned *myb80-like* male-sterile genotype, the adoption of a restriction enzyme that cuts depending on its exact sequence would provide a fast and reliable method for predicting male-fertile and male-sterile phenotypes before anthesis and even before flower development. With this aim, the Tru1I restriction enzyme was considered for its adoption in a CAPS marker assay able to distinguish the three possible genotypes and related phenotypes (two fertile: *MsMs* and *Msms*; one sterile: *msms*). Consequently, the investigation of associations between the GBS-derived RADtags and the CAPS results have been performed concerning male sterility identification, aiming for future GBS-based marker-assisted selection (MAS) protocols without the need for further experiments.

## 2. Results

### 2.1. RAD Sequencing Output

After the gDNA was extracted and evaluated and the 96 plant samples initially considered were sequenced, two of them were excluded due to a high number of missing data and sequencing quality results. The molecular data for the remaining 94 samples were retained for bioinformatic analyses.

RAD sequencing produced approximately 180 million total raw reads with an average of 1.9 million reads per sample. After quality assessment and adapter trimming, the obtained reads were used to create a catalogue of consensus loci and then used for variant calling as a reference, as described in Stevanato et al. [31]. After variant calling, a starting pool of 9351 SNPs were obtained, contained in 8918 RADtags. In a subsequent filtering step, the RADtags presenting at least one missing value among the population were discarded to increase the stringency of the analysis; the remaining 2953 SNPs, contained in 2917 RADtags, were used for the genetic statistics analyses to maintain proper representativeness.

### 2.2. Genetic Statistics and AMOVA

Regarding the genetic statistics computed for each population, the number of observed alleles ranged from 1.36 to 1.56 (na); the effective alleles (ne) were between 1.21 and 1.47. The observed (Ho) and expected (He) heterozygosity estimates were 12.09% and 11.93% minimum and 25.96% and 23.43% maximum, respectively, while the fixation index (F) ranged from 0.17 to −0.14. The percentages of polymorphic loci (PL%) and private alleles (PA%) were also computed and ranged from 56.48% to 36.23% for the first value and from 8.79% to 0.03% for the second value. All genetic statistics were calculated for each considered population and are reported in Table 1. The mean values were also computed for each statistic and are reported in Table 1.

Along with the genetic statistics for each population, the mean within-population expected (Hs) and total (Ht) heterozygosity, Wright’s F-statistics, and the gene flow (Nm) were also computed and reported in Table 2. Moreover, AMOVA was also computed to calculate the molecular variance within and among populations. The obtained results, shown in Table 3, indicate that 73.10% of the molecular variance is represented among populations and 26.90% within them. The computed probability is 0.001.

### 2.3. Genetic Similarity (GS) Estimates

From the GS analysis in all pairwise comparisons among the core collection, the resulting GS matrix (Supplementary Table S1) was used to compute the mean GS within and among each of the four populations represented in the core collection, together with the respective standard errors (±SE) (Table 4).

In detail, the genetic similarity ranged overall from 47.20% to 98.56% in single genotype estimations, with an average value of 65.53% among the core collection. The mean GS values within populations ranged from 82.33% (Pop1) to 91.05% (Pop4), while those among populations were between 54.08% (Pop3 vs. Pop4) and 76.60% (Pop1 vs. Pop2). Standard errors were also computed that were always |SE| < |0.05%| (Table 4).

**Figure 3:**
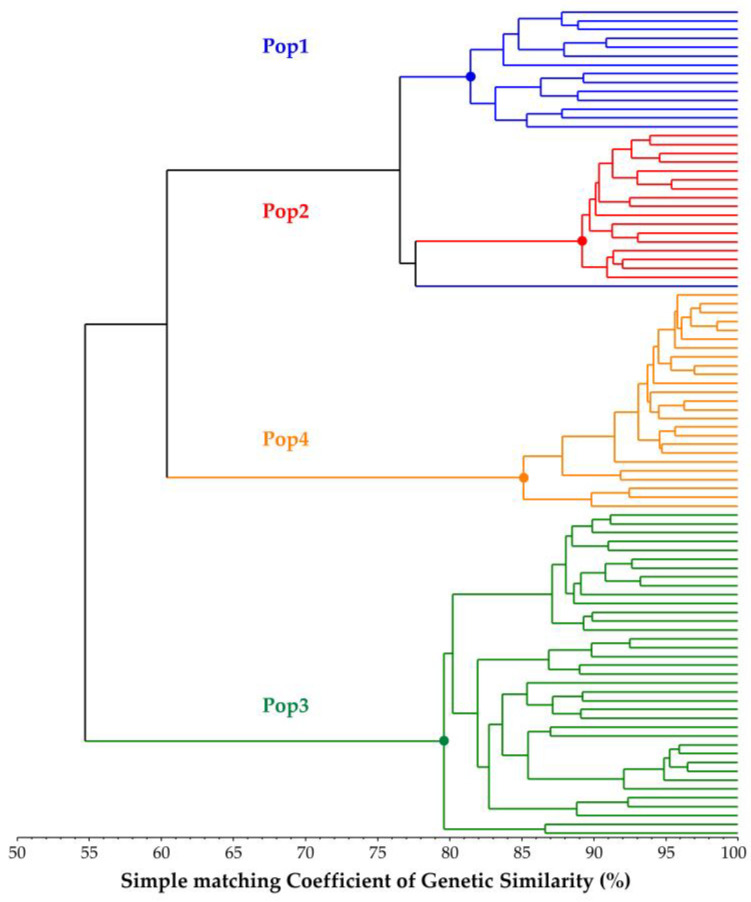
UPGMA dendrogram based on Rohlf’s simple matching coefficient of genetic similarity. Colours indicate the four different populations analysed in the core collection.

From the GS matrix obtained from the analysis of the 94 samples of the core collection successfully sequenced, a UPGMA dendrogram was created that grouped samples into four distinct branches according to the samples’ population of origin. One exception was observed for sample “Pop1-06” (labelled in blue), which was grouped closer to Cluster B (labelled in red), even though it was part of Pop1 (Figure 3).

Considering the results shown in the UPGMA dendrogram, a comparison between Pop3, coloured in green, and the other three populations, “Pop1”, “Pop2” and “Pop4”, was made that highlighted an observed GS of 54.66%, while an observed GS between the three populations located in one main branch of the dendrogram was 72.79%. For genetic similarity, observed homozygosity (Obs. Hom.) was also estimated, which presented the highest value in sample “Pop4-21” (Obs. Hom. = 94.48%), while the lowest was observed in sample “Pop1-03” (Obs. Hom. = 66.71%). Among the core collection, the mean observed homozygosity was 82.94%, while that within each population ranged from 74.04 ± 0.31% (Pop1) to 87.91 ± 0.13%.

**Figure 4:**
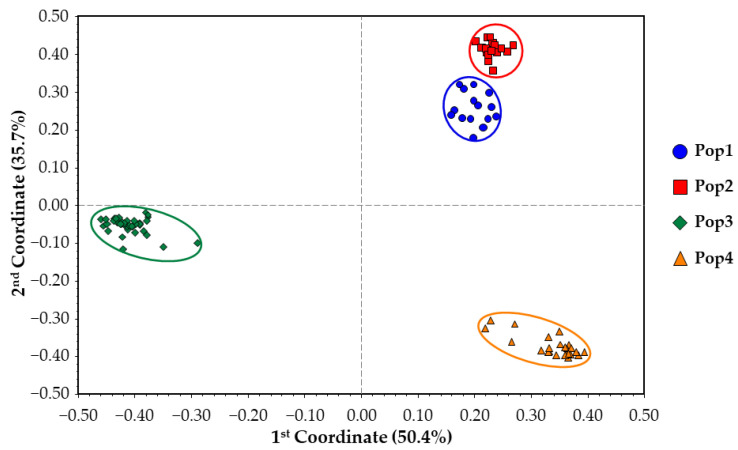
Principal coordinate analysis grouping the four chicory populations of the core collection into four distinct clusters according to samples belonging to populations and the clusters identified in the UPGMA dendrogram. Colour labelling is maintained from the dendrogram illustrated in Figure 3.

The GS matrix was also used to perform a principal coordinate analysis (PCoA) that clustered samples in the chart depending on Dimensions 1 and 2, which represented 50.4% and 35.7% of the molecular variability, respectively (86.1% in total) (Figure 4). This analysis was based on the eigenvectors calculated starting from the genetic similarity matrix and highlighted the same four clusters previously identified in the UPGMA dendrogram.

In general, the GS results highlighted that all four populations clustered independently from the others, although genetic relatedness was observed between Pop1 and Pop2. In contrast, Pop3 and Pop4 formed unique clusters with their respective individuals.

### 2.4. Genetic Structure Reconstruction of the Core Collection

Regarding the investigation of the genetic structure of the radicchio core collection, the results obtained from STRUCTURE software were then analysed using STRUCTURE HARVESTER web software to determine the most likely value of K depending on the ΔK values. The best result observed was K = 4 (∆K = 40,925) (Supplementary Figure S1), which was plotted as a histogram to represent the membership of each individual to one or multiple identified ancestral groups.

**Figure 5:**
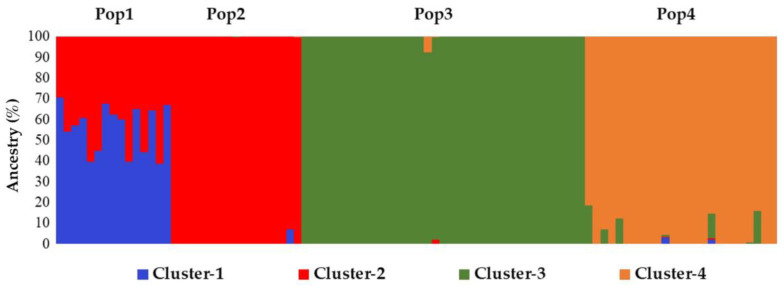
Histogram representing the STRUCTURE software computed results for K = 4. Different colour bars indicate the membership of each individual to a specifically labelled cluster, according to the same labelling adopted in the previous figures.

What emerged from this analysis was that among the four considered populations, three of them presented high membership values to a specific ancestral genotype (Pop2 to Cluster-2; Pop3 to Cluster-3; Pop4 to Cluster-4), while Pop1 was admixed with a membership ratio of 50:50 between the fourth group, Cluster-1, and Cluster-2 (Figure 5).

### 2.5. BLASTn-Based RADtag Mapping against Cichorium spp. Exomes

**Figure 6:**
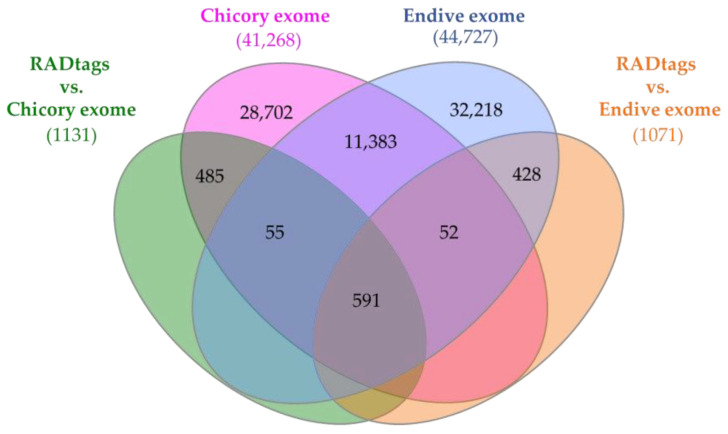
Venn diagram representing the BLASTn and BLASTx results of the matched CDSs of chicory and endive exomes and the congruences between these results and the CDS annotations obtained using the *L. sativa* exome as a reference.

**Figure 7:**
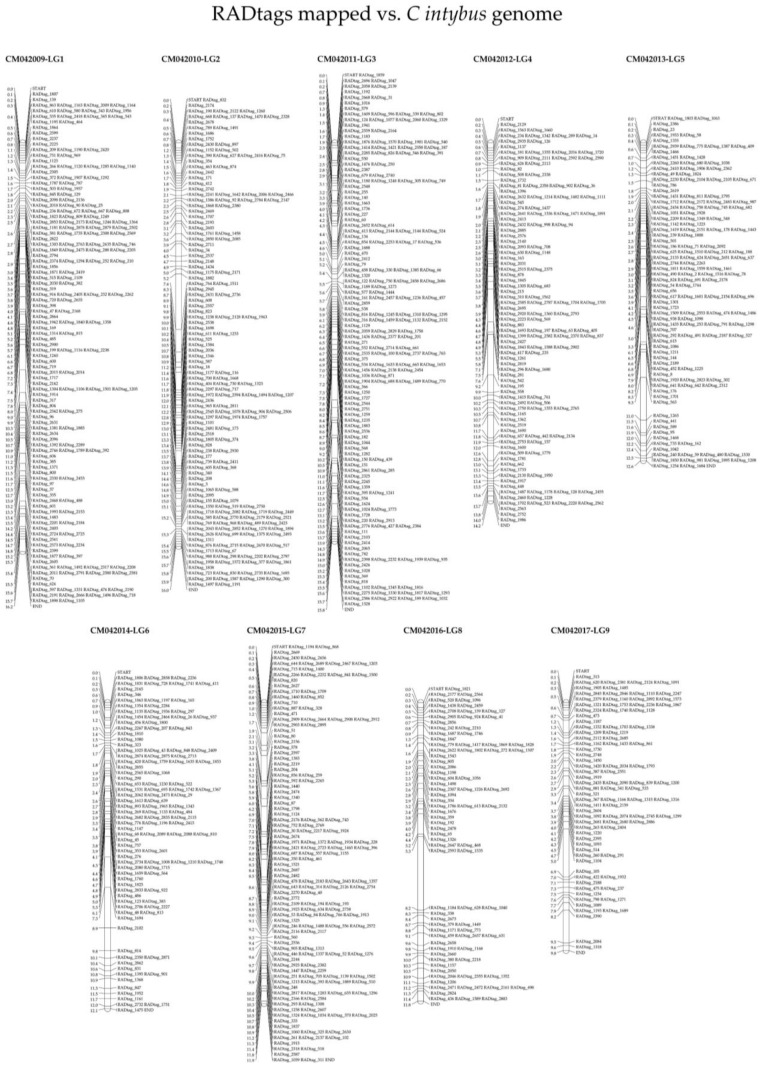
Chromosome representation of *C. intybus* L. RADtags matching CDSs among the nine assembled linkage groups (LG1 to LG9) are reported. Units on the left of each LG indicate 10,000,000 bps.

The BLASTx-based approach used to predictively name the “hypothetical protein” annotations of *C. intybus* and *C. endivia*, showed partial results. Specifically, the obtained results associate 40.16% (17,559 among 43,721 CDSs) and 31.13% (16,150 among 51,881 CDSs) of the chicory and endive CDSs with the *L. sativa* proteome (11,383 CDS annotations are shared between chicory and endive proteomes; Figure 6). As a consequence, the remaining annotations of the two *Cichorium* exomes remain named “hypothetical proteins” (Supplementary Tables S2 and S3). Given this, all 8918 RADtags obtained from RADseq were used in a preliminary BLASTn investigation against the 2 *Cichorium* species genomes that showed 5850 and 5404 RADtags matching the *C. intybus* and *C. endivia* genomes, respectively. Among the RADtags mapping the entire genomes of the *Cichorium* species, 740 and 294 were specific for chicory and endive, respectively. These results highlighted the presence of genome-specific RADtags. A further investigation was then conducted with the 2917 RADtags containing 2953 SNPs sequenced for all 94 samples (no missing RADtags among any sample) to map them against the 2 newly annotated *Cichorium* exomes. In particular, the use of CDSs, instead of entire genomic sequences, aimed to provide predictive information about putative polymorphic positions within coding regions. In total, 1308 and 1255 RADtags matched 1131 and 1071 CDSs with an average identity of 98.00% and 97.23% in *C. intybus* and *C. endivia*, respectively (BLASTn results for chicory and endive are available in Supplementary Tables S4 and S5). The results of the BLASTn and BLASTx analyses for chicory and endive are also reported in Figure 6, in which the shared results of the different computations are highlighted. In particular, 591 CDSs were successfully annotated using the *L. sativa* proteome and were also matched by RADtags in both chicory and endive, while 55 and 52 CDSs were annotated in both *Cichorium* exomes, although they were uniquely matched by RADtags in *C. intybus* and *C. endivia*. Moreover, RADtags matching exomes of chicory were plotted in the corresponding linkage groups and reported in Figure 7. Similarly, in Supplementary Figure S2 the same plotting was made for the results against the endive exome. This was made to give a graphical representation of the RADtag distribution across the genomes of the two species. The statistics of the BLASTn results are available in Table 5.

### 2.6. CAPS Assay and myb80-like/RADtag Association Investigation

In breeding programs exploiting male sterility, the characterisation of NMS locus genotype is of central importance both to maintain the male-sterile lines in the breeding population—by separating *Msms* heterozygous genotypes from the *msms* homozygous male-sterile ones—and to properly select the maternal plants for the F1 hybrid constitution. For this reason, practical and easy-to-use screening methods would be helpful.

**Figure 8:**
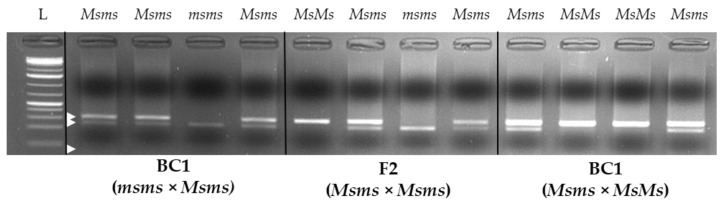
Agarose gel of the male-sterility characterization CAPS marker representing the obtainable bands (white arrows) in the case of the three different observable genotypes at the *Myb80-like* gene (phenotypes): *MsMs* (fertile), *Msms* (fertile) and *msms* (sterile). (L: molecular weight ladder; white arrows indicate the expected band sizes of 300, 230 and 70 bp, top to bottom).

From the testing experiment of the CAPS assay on samples of known sterile/fertile phenotypes, the obtained results showed complete agreement between the expected and observed results. Specifically, samples known as “fertile”, thus having the *Ms/–* genotype, always presented one or three bands after AGE, while those samples having a “sterile” phenotype always presented two bands, as expected and described in the “Methods” paragraph. As shown in Figure 8, 1 band at ~300 bp height indicates no restriction events by *Tru1*I enzyme, thus homozygous male-fertile genotype (*MsMs*), while in the case of 2 bands present at ~230 bp and ~70 bp, complete digestion of both homologous *myb80* alleles occurred (*msms*). Consequently, the presence of three bands in the AGE was the result of a partial restriction of the PCR products obtained, which reflects the heterozygous genotype *Msms*. Due to light exposure settings and the reduced size of the shorter restricted fragment, it is not always possible to identify the lowest band at ~70 bp.

**Figure 9:**
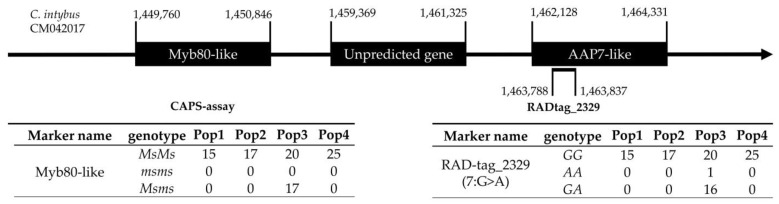
Graphic representation of RADtag_2329 in the *C. intybus* genome with the closest AAP7-like and unpredicted genes. Starting and ending positions for each annotation and the RADtag are reported following the sequence direction and expressed in base pairs. Genotype numbers for Myb80-like and RADtag_2329 are reported in the respective tables below the annotation.

After the evaluation of the CAPS assay efficiency and accuracy, a further investigation was performed to verify the presence of RADtags associated with the *Myb80-like* locus, which could be used in future studies as a predictive tool for determining the MS-locus genotype without using multiple experimental approaches. With this purpose, the results of the RADtag mapping analysis previously described have been used. From these results, it was possible to identify one RADtag matching close to the *Myb80-like* gene in the *C. intybus* genome. From our findings, RADtag_2329 (SNV: A/G; nucleotide sequence: 5′-TGCAGGCGGTTCACACCATTTTAGGTGAGGTTGTTTCATTTACGATTTAC-3′) was found within a “probable amino acid permease 7” (*AAP7-like*) coding sequence at 13 Kbps from the initial codon of *Myb80-like* in chicory (CM042017, region: 1,450,846 to 1,449,760). RADtag matching position and genomic region annotations are reported in Figure 9. Given these mapping results, the CAPS assay for identification of male sterility was performed on the 94 samples successfully sequenced. As it was known that none of the sequenced samples was male-sterile, the obtained results agreed with our expectation, and only male-fertile genotypes (*Ms*/–) have been identified. In particular, the only population presenting heterozygous genotypes was Pop3, in which one disagreement between the *MsG/msA* association was observed, as one individual presented the *Msms* genotype at locus *Myb80*-*like* and the *AA* genotype for RADtag_2329. Considering that one mismatch was observed among the 94 genotyped samples (1.06%), this result has been attributed to a sequencing or raw read merging error. Data are reported in Figure 9.

### 2.7. Predicting Progeny Genotypes in Planned Selfing, Full-Sibling and Pairwise Crossing Mating Strategies

**Figure 10:**
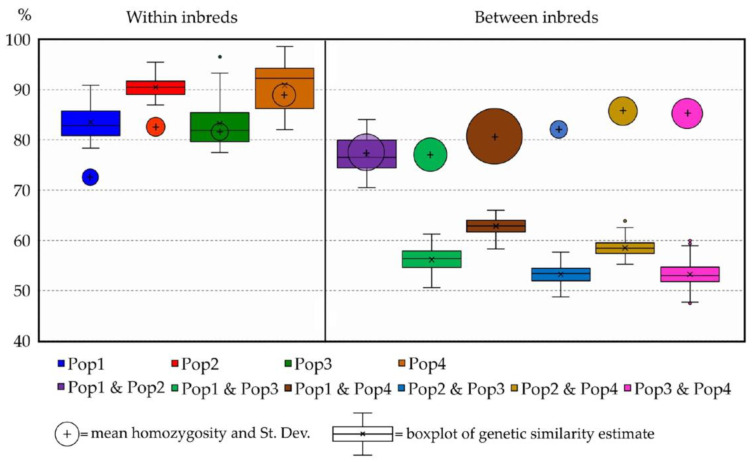
Boxplot of the mean genetic similarity among the 40 selected suitable parental individuals calculated within populations (left side) and among populations (right side). Bubble position in the chart indicates mean homozygosity, while their size reflects the respective standard deviation. In addition to the maximum and minimum values, the second and third quartiles of each boxplot are marked inside each square and are divided by the median bar, while the mean value is represented by the cross (×). Dots indicate outlier samples.

**Figure 11:**
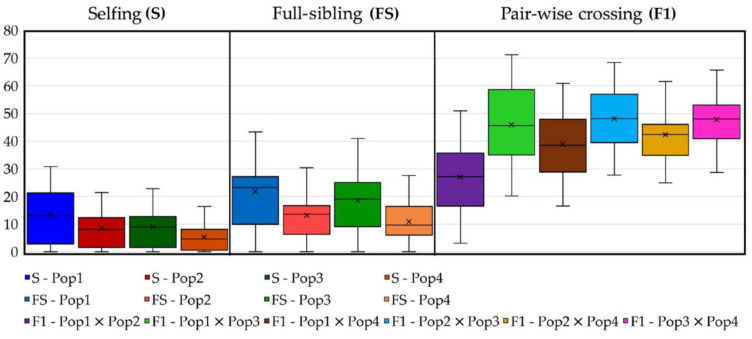
Boxplot of heterozygosity estimates referred to progenies obtained by selfing (S), full-sibling (FS) and pairwise crossing (F1) strategies using genotype information of the 40 selected analysed individuals. In addition to the maximum and minimum values, the second and third quartiles of each boxplot are marked inside each square and are divided by the median bar, while the mean value is represented by the cross (×).

Consistent with the main aim of this study, concerning the identification of putative pairwise crossing, selfing or full-sibling mating strategies for breeding purposes, heterozygosity values of the putative progenies were computed, as described in the “Methods” below, in which 10 putative parentals were selected from each of the analysed populations. As reported in Supplementary Table S6, the quadrangular matrix 40 × 40 presents the genetic similarity values in all pairwise comparisons among the 40 selected parental individuals (below diagonal) and the expected progeny average heterozygosity values (above diagonal). Regarding the chosen parentals, the genotype homozygosity and heterozygosity are reported (as in Supplementary Table S1). What emerges from this computation is that, in the case of high genetic similarity between parental individuals, the computed progeny heterozygosity results are very low, as expected (selfing and full-sibling mating); however, in the pairwise crossing-derived progenies, heterozygosity is increased compared to the parentals and, as hypothesised, the best pair-wise crossing results can be obtained from the most dissimilar and homozygous parentals. In Figure 10, the GS values computed within and among the 40 selected parentals are shown in combination with their respective homozygosity, which is represented as bubbles differing in size depending on the calculated standard deviation. In parallel, the plotting of the expected heterozygosity values of the hypothetical progenies is reported in Figure 11, where selfing-derived values of heterozygosity are shown to always be lower than those of the full-sibling progenies (especially in slightly uniform parental populations such as Pop1) and much lower than those of the pairwise crossing. One particular exception is represented by the pairwise crossing between Pop1 and Pop2, in which the plotted results are comparable with those obtained in the hypothetical full-sibling.

## 3. Discussion

RADseq (restriction site-associated DNA sequencing) is a relatively new technology that has revolutionised plant genotyping. The cost-effectiveness of RADseq and its high-throughput genotyping data in studies involving large numbers of individuals and populations showed the many possible applications of this method in plant population genetics and plant breeding programs. Currently, this technology is extensively adopted in plant genomics to map and implement molecular markers associated with desirable traits and to develop new varieties with improved agronomic performance.

RADseq has been used in many plant species, including crop plants such as maize, wheat and rice, wild tomato accessions and model systems such as Arabidopsis. Some of the first references for the use of RADseq in plant genotyping include “Genotyping-by-sequencing for plant breeding and genetics” by Poland and Rife [25], and “Genotyping-by-sequencing approaches to characterize crop genomes: choosing the right tool for the right application” by Scheben et al. [34].

Additional and previously conducted research studies have reported the use of RADseq as a reliable tool for genotyping crop species [31,32,35,36]. Genotyping-by-sequencing through RADseq and SNP markers have many possible applications for breeders in MAB, MAS or PVP purposes. In Chen et al. [37], the use of RADseq allowed the selection of drought-tolerant soybean varieties through genome-wide association studies and marker-assisted selection. Additionally, RADseq genotyping data were useful in lettuce for identifying molecular markers associated with pest resistance traits to Fusarium wilt race 2 [38]; a serious cause of yield losses; in soybean; to identify QTLs associated with yield and oil content that could be exploited in MAB strategies through MAB [39]; and in tomato to reconstruct the population structure of cultivated tomato lines, providing new insights into the Mediterranean long shelf-life germplasm [40], which could be used to develop new tomato varieties with improved characteristics. In comparison to other molecular PCR-based approaches, the advantages of RADseq consist of having not only the genotype information but also the availability of nucleotide sequences for each of the marker loci analysed, which can then be mapped in the own genome of the species of interest or in those of related ones [41,42,43], which provides valuable knowledge for developing fast and efficient targeted PCR-based analytical assays. Moreover, given the high data throughput of the method adopted (1.8 million raw reads per sample; 9351 SNVs and more than 8 thousand RADtags, in the present study), the identification of multiple discriminant alleles for different phenotypes would improve MAS approaches for Mendelian genes and polygenes, or QTLs, by combining multiple information in a single experiment [27]. These aspects, combined with the reduction in the analysis cost per sample and compared to PCR-based assays [44], make RADseq a suitable and effective analytical tool for both genetics and breeding, as well as basic research applications. However, it is worth mentioning that fast, inexpensive and informative whole-genome screening assays based on RADseq technology may also be needed in the frame of plant breeding programs for MAB strategies, aimed at assessing homozygosity/heterozygosity estimates as well as genetic uniformity within and genetic differentiation between parental inbred lines.

RADseq technology has been used in many studies for genotyping different crop species. It is noteworthy that, in the current scientific literature, the only case study in which RADseq technology is used for genotyping *Cichorium* species is the work of Patella et al., in which endive accessions were analysed using RADseq to identify the genes responsible for leaf shape in this crop [45]. Moreover, germplasm characterisation and population genetics studies based on RADseq genotyping have been performed, which showed the potential of this tool in other crop plants, such as peanut [46] and faba bean [47], as well as in other non-plant organisms. An example of this is represented in Sunde et al., where a comparison between codominant PCR-based microsatellite and RADseq is also provided [48], highlighting the higher informativeness of the GBS approach compared to conventional PCR.

Based on our previous endive study [45], RADseq has proven its suitability in a related species such as chicory in the present study. Here, we successfully analysed 94 individuals belonging to 4 distinct populations of chicory. The genetic statistics, based on almost 3000 SNP markers, demonstrated the good informativeness of the provided method. The ne, which is slightly lower than na, as expected, reflects a relevant variability (total ne = 1.32; total na = 1.46) within the observed populations and overall. In agreement with these findings, the F value was close to or below zero among the four populations, thus indicating random mating within them, which is consistent with the full-sibling nature of the analysed populations. Moreover, PL% indicates a relevant variability of both the molecular markers used in the analysis and the analysed populations. Notably, the observed number of private alleles (%PA_max_ = 8.79% in Pop3 and %PA_total_ = 16.00%) reflects variable distinctiveness within and among the four populations analysed, which is important information that can be used for PVP and traceability purposes. Other findings that indicated the genetic variability within populations are the observed heterozygosity (Ho_tot_ = 17.89%) and the F-statistics reported in Table 2. Notably, gene flow (Nm = 0.631) [49,50] indicates a tendency of the population to inbreed, in agreement with the full-sibling nature of the populations analysed. Another result, obtained through the AMOVA, reports that almost 75% of the total molecular variation occurs among populations (Table 3), which, combined with PA values and the other statistics already discussed, suggests a good distinctiveness of the four single FS lines used in this study, as well as a relatively high genetic uniformity of each population (82.33% < within GS < 91.05%). Moreover, as a confirmation of this hypothesis, both the results of the among-population GS and genetic structure separate individuals into four distinct clusters reflecting the core collection division into the four populations. Each of the individuals of the core collection are associated with its original population, but, as suggested by the UPGMA dendrogram (Figure 3), individuals from Pop1 and Pop2 are closely related. Additional information sustaining these findings was obtained from the STRUCTURE analysis, in which Pop1 was admixed with a 50% membership on average with Pop2’s Cluster-2 (Figure 5), thus suggesting that Pop1 is probably derived from Pop2. Moreover, the homozygosity results, combined with the within GS calculated for each population, highlighted different uniformity degrees, from highly uniform and homozygous populations (Pop4) to less uniform and heterozygous ones (Pop1). In this regard, the predictive analysis of the potentially obtainable progenies from selfing, full-sibling or pairwise crossing the best parental individuals selected from each population starting from the RADseq characterization highlights that parentals presenting high levels of homozygosity and within GS can be preferentially adopted for the constitution of F1 hybrid lines (e.g., Pop3 × Pop4), while those that do not match these requirements are more indicated for homozygosity increments, although through self- or sibling-mating strategies (e.g., Pop1). Moreover, highly related parentals, even those belonging to different populations, make cross-pollinations undesirable for constituting F1 hybrids due to the low obtainable heterozygosity, as demonstrated by the expected He (e.g., Pop1 × Pop2) (Figure 10 and Figure 11).

As this study aims to provide a pipeline suitable for MAB, MAS and PVP, not only the possibility of overall genotypic characterization based on genome-wide platforms but also the locus-specific characterization of molecular markers related to specific traits is important. In this regard, the locus association between the RADtag_2329 and *Myb80-like* genomic regions is a possible example. Male sterility predictive information can be adopted in future screening analyses aimed at the development of F1 hybrid cultivars by means of pair-wise crosses with highly differentiated pollen donors to maximize hybridity and heterozygosity, and thus to exploit the potential manifestation of heterosis. In fact, from the identification of the *msms* genotypes, seed parental lines or clones can be selected to be crossed using fertile genotypes (*MsMs*) as pollinators. This strategy prevents maternal plants from developing inbred seeds, characterised by higher homozygosity, thus favouring the production of only F1 hybrid seeds derived from cross-pollination strategies, which have higher heterozygosity and potential heterotic vigour [17]. Parallel to this first promising result, the same goal can be achieved for other RADtags. The potential of RADseq methodology in identifying molecular markers associated with specific genes involved in phenotypic traits of agronomic interest has been highlighted by the RADtag mapping analysis results, consisting of 1084 matches with both *C. intybus* and *C. endivia* exomes, which can be investigated in future studies for the validation of suitable molecular markers for MAS approaches. An additional insight related to these results is that the BLASTn analysis against the newly available genomes of chicory and endive reported 5110 RADtags (also considering those with missing values among one or more samples) shared among the 2 genomes, while 740 and 294 were specific for *C. intybus* and *C. endivia*, thus supporting the relatedness to these 2 species and the interspecific nature of the “Red of Chioggia” biotype to which all tested samples belong. This latter information provides useful knowledge for several purposes: interspecific hybrid identification, and phylogenesis and evolution studies of these two related crop species. Moreover, the possibility of investigating which of the identified SNPs were contained in expressed genomic regions (1308 and 1255 in chicory and endive, respectively) could provide a suitable starting point to develop future screening tools for MAS strategies in both C*ichorium* species, as well as genetic traceability tools for protecting cultivars from frauds or mislabelling (PVP). Another application in which this information can be used is the identification of suitable genetic resources to be introduced or genetic traits to be introgressed by specific breeding programs for each of these two crop plants, aimed at improving their environmental adaptability, resistance or tolerance to pests and morphological characteristics of agronomic and commercial value.

In conclusion, our study demonstrates that RADseq is a powerful tool for genotyping plant materials of leaf chicory, providing accurate estimates of homozygosity and heterozygosity, genetic similarity and diversity statistics that can be used for planning breeding programs aimed at the development of inbred lines and F1 hybrids in radicchio.

## 4. Materials and Methods

### 4.1. Plant Materials

In total, 4 full-sibling (FS) populations of Italian red chicory, biotype “Red of Chioggia”, for a total number of 96 individuals, were used in this study for RADseq-based genotyping. Populations belonging to separated inbred lines were selected to verify the homozygosity and genetic similarity within and among them, respectively. Genomic DNA was extracted for each sample using 100 mg of fresh leaf tissue using the DNeasy^®^ 96 Plant Kit (Qiagen, Valencia, CA, USA) and following the protocols provided by the manufacturer. After DNA extraction and purification, DNA quality, quantity and integrity were evaluated using a NanoDrop 2000c UV-Vis spectrophotometer (Thermo Fisher Scientific Inc., Pittsburgh, PA, USA), and agarose gel electrophoresis was prepared as a 1% agarose/1× TAE gel containing 1× Sybr Safe DNA stain (Life Technologies, Carlsbad, CA, USA). Moreover, the genomic DNA purification, quantification and quality evaluation protocols described above were also adopted to isolate the gDNA of 12 samples of known fertile/sterile phenotypes derived from 3 different segregating populations to be used for CAPS assay validation. Regarding the origin of these samples, four individuals belonging to a BC1 line with only heterozygous (*Msms*) and sterile homozygous (*msms*) individuals, four individuals from an F2 line with all three possible genotypes represented and four individuals from a fertile BC1 population with heterozygous and fertile homozygous (*MsMs*) genotypes were adopted.

### 4.2. RADseq Analysis and Data Analysis

Among the 96 gDNA samples obtained, 94 were successfully sequenced through the restriction site-associated DNA sequencing (RADseq) approach. Due to the high number of missing data, two samples were not considered in the analyses. RADseq analysis was performed using 1 μg of gDNA per sample and restricted using *MseI* enzyme (New England Biolabs, Ipswich, MA, USA); the procedure is described by Stevanato et al. [31]. For library preparation, digested DNA samples were diluted to a concentration of 3 ng/μL, and indexing, library preparation, sequencing and bioinformatic analyses of the raw reads were performed according to the protocol described by Stevanato et al. [31]. Raw reads obtained through an Ion S5 sequencer (Thermo Fisher Scientific Inc., Waltham, MA, USA) were trimmed according to the restriction enzyme recognition motif. After quality assessment, all artefacts and Ns-containing reads were removed, and nucleotide variants were assessed using Stacks v2.41 software [51]. SNPs with a sequence depth < 4× and with more than 2 allelic variants were filtered out. Only biallelic SNPs were considered in this study.

### 4.3. Data Analysis and Genetic Statistics

The obtained biallelic molecular marker data were analysed to calculate genetic statistics. The genetic similarity (GS) was computed using Rohlf’s simple matching coefficient in all pairwise comparisons, and the resulting GS matrix was used for the construction of an unweighted pair group method with arithmetic mean (UPGMA) dendrogram and a principal coordinate analysis (PCoA) using NTSys software v2.21 (Applied Biostatistics Inc., Port Jefferson, NY, USA) [52]. Moreover, the GenAlEx 6.5 [53] Excel macro was used to calculate the number of observed and effective alleles, the observed and expected heterozygosity estimates, the fixation index, the percentage of polymorphic loci, the number and percentage of private alleles of each population and identified overall, Wright’s F statistics, heterozygosity estimates and gene flow. AMOVA was also computed using GenAlEx software. Alongside the genetic statistics and similarity estimates, a Bayesian clustering algorithm implemented in STRUCTURE v.2.2 [54] was used to reconstruct the genetic structure of the core collection. The parameters adopted in this analysis consisted of numbers of founding groups ranging from 1 to 20, and 10 replicate simulations were conducted for each value of K based on a burn-in of 200,000 and a final run of 1,000,000 Markov Chain Monte Carlo (MCMC) steps. STRUCTURE HARVESTER [55] was used to estimate the most likely value of K based on the STRUCTURE software analysis results, and the estimates of membership were plotted as a histogram using an Excel spreadsheet.

### 4.4. BLASTn Analysis and RADtag Mapping

After the genetic statistics analysis, a further investigation was performed to verify which identified SNPs belonged to coding regions of the chicory genome.

Considering the newly published genomic assembly for both chicory (*C. intybus* L.) and endive (*C. endivia* L.), we considered these two datasets for the following BLASTn analysis (BLAST+ 2.11.0 package). The two assemblies were retrieved from NCBI using the accession numbers GCA_023525715.1 (chicory) and GCA_023376185.1 (endive). Although the two assemblies presented putative coding sequence (CDS) annotations (51,881 and 43,721 for endive and chicory, respectively), these were only reported as “hypothetical proteins” with no information about their genic function or gene ontology with other species. For this reason, and because our intention was the identification of the CDS-matching RADtags with possible phenotypic interaction/effect, further analysis was needed to putatively assess the nature of the CDS annotations. For this purpose, the translated exome of lettuce (*Lactuca sativa* L.; NCBI accession number: GCF_002870075.3) was adopted in a preliminary BLASTx analysis to annotate the two *Cichorium* CDSs using their translation as queries. For this analysis, UseGalaxy [56] implementing BLASTx (BLAST+ 2.11.0 package) software was used in two parallel computations for the two species considered.

After the *Cichorium* species genome annotation, two parallel BLASTn analyses were conducted to map the RADtags presenting no missing information among the core collection against the newly annotated CDSs. All obtained RADtags, both the completely shared and those missing in at least one genotype, were used in two BLASTn analyses against the entire genomic sequences (intra- and extragenic). In detail, the parameters adopted in these steps followed those described in Scariolo et al. [57], in which the following parameters were used: an E-value threshold ≤ 1.0 × 10^−10^ and a percentage of identity ≥ 80%, plus a minimum query coverage of 80%.

RADtag mapping and BLASTn analysis results were plotted in a Venn diagram using InteractiVenn web software [58] to highlight the shared CDSs annotated through *L. sativa* exome support, the CDSs shared between two exomes of the *Cichorium* species, plus the RADtag matches that were shared between the two reference exomes and those that were specific for one or the other. Moreover, two linkage maps have been created presenting *C. intybus* and *C. endivia* linkage groups that report the mapping region of the RADtags matching CDS in the exomes of the two species. Linkage maps were created using MapChart 2.32 [59]

### 4.5. Cross-Strategy Planning for Breeding Purposes

Considering the genotypes characterised through the RADseq approach, the GS estimates in all pairwise comparisons among the 94 samples successfully sequenced and analysed, as well as their observed homozygosity values, a further investigation of the most suitable crosses for different breeding purposes was performed. Specifically, the genotype information of the 10 selected individuals for each of the 4 populations was used to hypothesise the genetic estimates obtainable in putative progenies derived from selfing, full-sibling (within the population) and pairwise crossing (between different populations) breeding strategies. In particular, the possible application and aims of this investigation could be the obtainment of highly heterozygous and uniform F1 hybrids or the increment of homozygosity and uniformity within the populations considered in this study. To investigate the possible application of the GBS-derived information, the 40 selected genotypes were chosen for presenting high GS with individuals from the same population (within GS > 90%, when possible) and the highest homozygosity. This was made to simulate the mating scenarios of inbreeding, aimed at obtaining highly uniform and homozygous putative parental lines, and out-breeding for developing F1 hybrids. Considering that the molecular markers analysed in this study are biallelic SNPs, the canonical Mendelian gene recombination expected frequencies were adopted for this computation, although the loci association was not considered as no information is available yet about the most common regions of crossing-over in chicory.

In Table 6, the expected heterozygosity frequency values associated with each genotype × genotype crossing combination are reported. By adopting the information reported in Table 6, the He of all pairwise crossing combinations was computed for each locus in the case of selfing, full-sibling or pairwise crossing breeding strategies.

### 4.6. CAPS Screening Assay for Male-Sterility Identification

For the development of the CAPS assay for the characterisation of male sterility in chicory, research from Palumbo et al. has been followed. Two specific primers were designed by Primer3 software, using as references the myb80 sequences retrieved from NCBI and published by Palumbo et al. [11] under the accession numbers MK285054.1 (sterile phenotype) and MK285053.1 (fertile phenotype). The designed primers were named CiMyb80_for (sequence: ACTGCGGTTGCTGGTCA) and CiMyb80_rev (sequence: CCCTGCTCATGCTCCTG) and were used for the amplification of a 302 bp long sequence containing a palindromic insertion (when present) of 4 nucleotides (AATT) that is responsible for the coding frameshift that causes the male-sterile phenotype. The initial primer testing phase was performed using the following protocol: gDNA amplifications were performed using a Veriti 96-Well Thermal Cycler (Applied Biosystems, Foster City, CA, USA) in a total volume of 20 μL using 1× Platinum Multiplex PCR Master Mix (Thermo Scientific, Carlsbad, CA, USA), 5% GC Enhancer (Thermo Scientific), 0.25 μM of each primer, 30 ng of gDNA and sterile water to volume; the PCR thermal conditions were as follows: 5′ at 95 °C; 35 cycles at 95 °C for 30″, 56 °C for 30″, and 72 °C for 45″; and a final extension at 72 °C for 10′. After the PCR step, the obtained amplicons were digested using the Tru1I restriction enzyme (Thermo Scientific) that recognises the 5′-AATT-3′ nucleotide motif that exactly matches the 4 nt insertion in the male-sterile allele (*ms*). Restriction reactions were performed adopting the following procedure: 10 µL of PCR product, 1× Buffer R, 3 U/µL of Tru1I restriction enzyme, and sterile water up to 30 µL final volume. Restriction reactions were performed using a Veriti^®^ 96-Well Thermal Cycler (Applied Biosystems, Foster City, CA, USA) for 16 h (overnight) at 65 °C. After amplicon restriction, digested products were evaluated by agarose gel electrophoresis (AGE) using 2% agarose/1 × TAE gels containing 1 × SYBR Safe DNA Gel Stain (Life Technologies) to separate the eventually restricted amplicon patterns. Specifically, undigested (1 band at 300 bp height—*MsMs* homozygous male-fertile genotype), partially digested (3 bands at 300 bp, 230 bp and 70 bp heights—*Msms* heterozygous male-fertile genotype) and completely restricted amplicons (2 bands at 230 bp and 70 bp heights—*msms* homozygous recessive male-sterile genotype) were expected. Samples used in the validation of the method described here were selected for having a known phenotype (*Ms/–*: fertile*; msms*: sterile) and genealogy.

## 5. Conclusions

To conclude, the genotyping method adopted in this study has demonstrated its informativeness and discriminative ability in distinguishing populations belonging to the same biotype and in assessing genetic relationships among closely related breeding lines. Our results demonstrated that RADseq is a suitable approach for reconstructing the genetic structure of a breeding core collection of chicory interspecific hybrids, highlighting genomic information about the ancestral genotypes of the different investigated lines. Moreover, through the use of assembled genomes of related species,, comparative mapping analyses showed the potential of the data obtainable through RADseq, which can be adopted to exploit new genetic resources for breeding and traceability purposes, as already demonstrated in other studies [16,32,60,61]. In addition, the newly developed CAPS marker for male sterility characterisation has proven its reliability and possible applications in breeding planning, as well as its associated RADtag_2329, which is a unique marker that could be used in future wide-spectrum GBS approaches in this species.

Further studies are needed to verify the informative and predictive potential of RAD sequencing in this crop, especially focusing on genetically discriminant and phenotype-related markers to increase the applied exploitation of this genomic tool. Nonetheless, the obtained results are encouraging for the possibility of developing high-throughput MAS and MAB screening assays to be adopted in next-generation breeding approaches. In addition to breeding aims, RADseq has another potential use related to the reconstruction of phylogenies and characterisation of interspecific hybrids [62,63,64], which can provide informative knowledge about the origin of agronomically important biotypes of radicchio including the “Variegated of Castelfranco” and the “Red of Chioggia”. From the overall genomic knowledge gathered on these two main aspects, new intraspecific and interspecific crosses could be designed and performed to breed plant novelties that manifest phenotypic traits of interest, responding to current consumer needs and modern cultivation requirements.

## Figures and Tables

**Table 1:** Genetic statistics calculated for each population and mean overall. Reported information is the number of individuals (N), number of observed (na) and expected (ne) alleles, observed (Ho) and expected (He) heterozygosity percentages, fixation index (F), and percentage of polymorphic loci (PL).

Pop ID	N	na	ne	Ho (%)	He (%)	F	PL (%)
Pop1	15.00	1.56	1.43	25.96	23.43	−0.10	56.48
Pop2	17.00	1.36	1.27	16.33	14.02	−0.14	36.23
Pop3	37.00	1.54	1.38	17.17	20.77	0.17	54.01
Pop4	25.00	1.37	1.21	12.09	11.93	0.07	37.45
Mean	23.50	1.46	1.32	17.89	17.54	0.01	46.05

**Table 2:** Mean within-population expected heterozygosity (Hs), total heterozygosity (Ht), Wright’s F-statistics (Fis; Fit; Fst) and gene flow (Nm) calculated among the core collection. Standard errors (SEs) are also reported for each value.

	Hs	Ht	Fis	Fit	Fst	Nm
Total	0.18	0.36	0.03	0.48	0.48	0.63
SE	0.00	0.00	0.01	0.01	0.01	0.03

**Table 3:** AMOVA results. Degrees of freedom (df), sum of squares (SS), means squares (MS), and estimated variance (Est. Var.) with the respective percentage (%) are reported.

Source	df	SS	MS	Est. Var.	%
Among Pops	3	104,231	34,743	1523	73.10
Within Pops	90	50,439	560	560	26.90
Total	93	154,671		2083	100.00

**Table 4:** Observed homozygosity (%) and mean pairwise genetic similarity matrix calculated within and among the four radicchio populations; standard errors are also reported.

Obs. Homozygosity (%)	Population ID	Mean Genetic Similarity (%)			
74.04 ± 0.31	Pop1	82.33 ± 0.04			
83.67 ± 0.15	Pop2	76.60 ± 0.01	90.12 ± 0.01		
82.83 ± 0.13	Pop3	56.46 ± 0.00	54.13 ± 0.00	82.50 ± 0.01	
87.91 ± 0.13	Pop4	62.59 ± 0.01	58.46 ± 0.00	54.08 ± 0.00	91.05 ± 0.01
		Pop1	Pop2	Pop3	Pop4

**Table 5:** BLASTn analysis results of the no-missing-data-containing RADtags against the *C. intybus* and *C. endivia* exomes. Numbers of matching RADtags, CDSs, average mismatches (Avg. mis), average identical positions (Avg. ident) and exome-specific RADtags (Ex. Spec.) are reported alongside the percentages of average identity (Avg. ident), the average length of the alignments (Avg. length), exome-specific RADtags (Ex. Spec. %) and shared RADtags. The supporting statistics, such as E-value, bit score (Bs) and score (score), are also reported as average values for both analyses.

BLASTn Results	RADtags (*n*)	CDS (*n*)	Avg. Ident (*%*)	Avg. Ident (*n*)	Avg. Length (*bp*)	Avg. Mis (*n*)	Avg. E-Value	Avg. Bs	Avg. Score	Ex. Spec. (*n*)	Ex. Spec. (%)	Shared (*n/%*)
*C. intybus* exome	1308	1131	98.00	62.22	63.50	1.26	9.04 × 10^−13^	109.60	120.55	224	15.15	1084 / 73.29%
*C. endive* exome	1255	1071	97.23	61.91	63.70	1.74	4.24 × 10^−13^	107.73	118.43	171	11.56	

**Table 6:** Parental genotype × genotype crossing combination and relative expected average heterozygosity (He) in the progeny.

P1 Genotype *	P2 Genotype *	P1 × P2 Average He
0	0	0
1	1	0
0	1	1
1	0	1
0/1	2	0.5
2	0/1	0.5
2	2	0.5

* 0: homozygous for allele A; 1: homozygous for allele B; 2: heterozygous.

## Data Availability

Not applicable.

## Supplementary Materials

The following supporting information can be downloaded at: https://www.mdpi.com/article/10.3390/plants12061242/s1: Figure S1: Graph representing the ∆K values resulting from Structure Harvester software analysis of STRUCTURE software results; Figure S2: Chromosome representation of *C. endivia* L. RADtags matching CDS among the nine assembled linkage groups (LG1 to LG9) are reported; Table S1: Genetic similarity matrix (94 × 94) in all pairwise comparisons; homozygosity (Ho) and heterozygosity (Ho) are reported for each genotype; Table S2: BLASTx results of “hypothetical” annotated *C. intybus* proteome against *L. sativa* proteome; Table S3: BLASTx results of “hypothetical” annotated *C. endivia* proteome against *L. sativa* proteome; Table S4: BLASTn results of RADtags against annotated *C. intybus* exome; Table S5: BLASTn results of RADtags against annotated *C. endivia* exome; Table S6: Quadrangular matrix 40 × 40 presenting the GS values in all pairwise comparisons among the 40 selected parental individuals (below diagonal), the expected progeny average heterozygosity values (above diagonal) and the parental individual’s homozygosity and heterozygosity (two columns left).
