# Supplementary material for: Pipeline to Design Inbred Lines and F1 Hybrids of Leaf Chicory (Radicchio) Using Male Sterility and Genotyping-by-Sequencing
Source: Plants (Basel). 2023 Mar 9;12(6):1242. doi: 10.3390/plants12061242 (PMC10055022; doi:10.3390/plants12061242)
Supplement: Supplementary file 1 [file plants-12-01242-s001.zip › RADseq_CAPS_radicchio_Supplementary_Figures.pptx]

## Slide 1
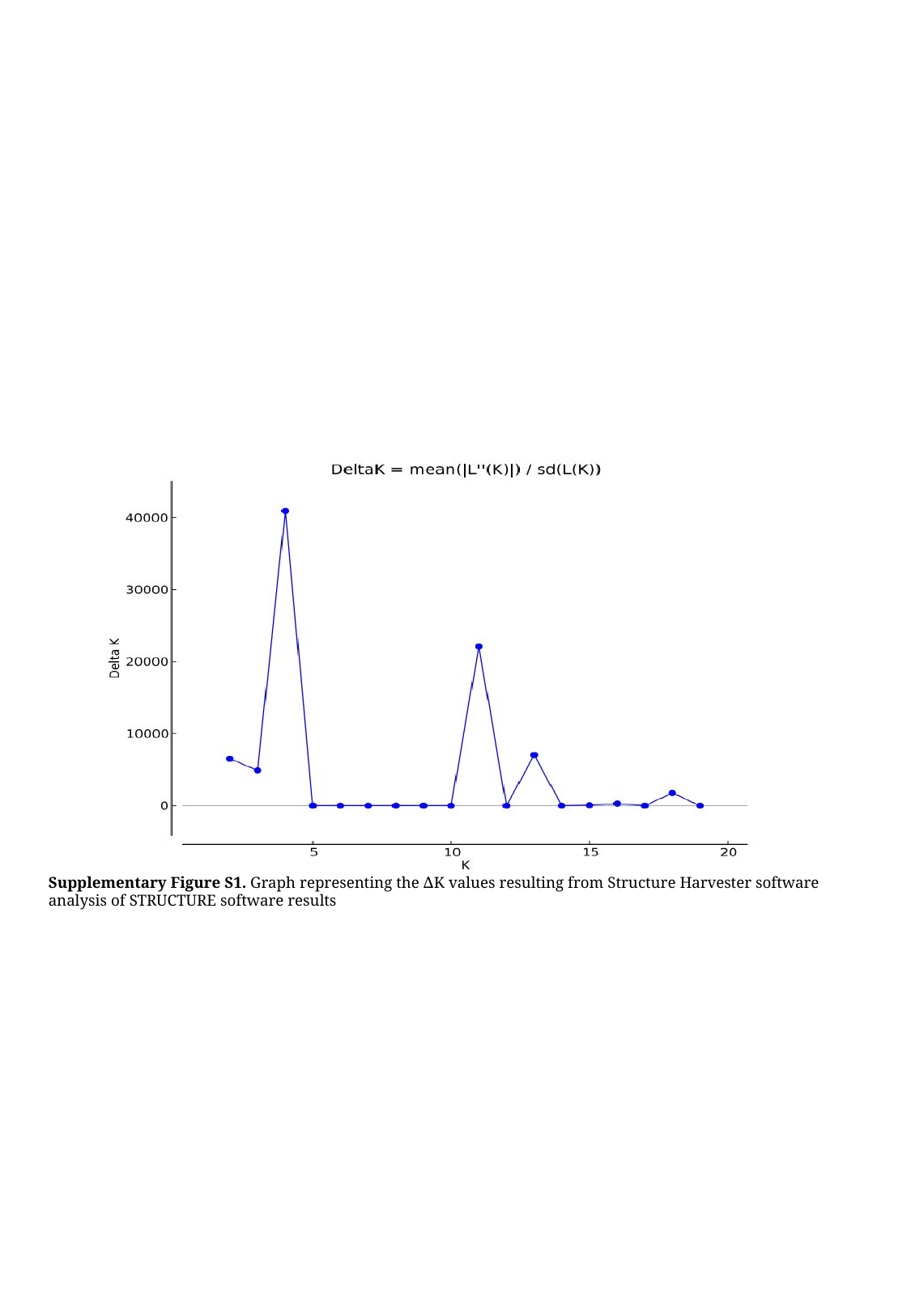

Supplementary Figure S1. Graph representing the ∆K values resulting from Structure Harvester software analysis of STRUCTURE software results

## Slide 2
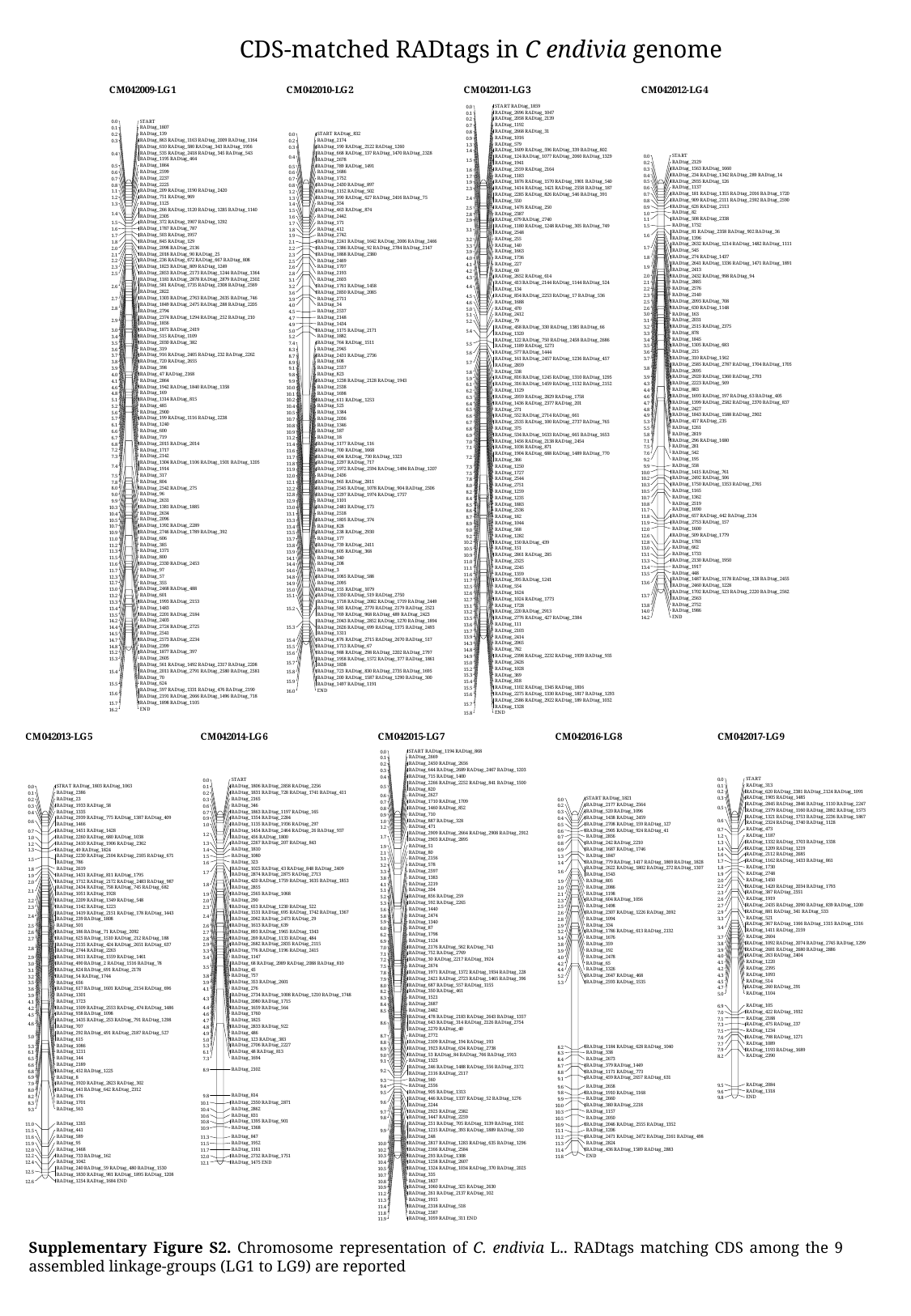

CDS-matched RADtags in C endivia genome
Supplementary Figure S2. Chromosome representation of C. endivia L.. RADtags matching CDS among the 9 assembled linkage-groups (LG1 to LG9) are reported
